# Predictive Mapping of Antimicrobial Resistance for *Escherichia coli*, *Salmonella,* and *Campylobacter* in Food-Producing Animals, Europe, 2000–2021

**DOI:** 10.3201/eid3001.221450

**Published:** 2024-01

**Authors:** Ranya Mulchandani, Cheng Zhao, Katie Tiseo, João Pires, Thomas P. Van Boeckel

**Affiliations:** ETH Zürich, Zurich, Switzerland (R. Mulchandani, C. Zhao, K. Tiseo, J. Pires, T.P. Van Boeckel);; One Health Trust, Washington, DC, USA (T.P. Van Boeckel)

**Keywords:** Antimicrobial resistance, maps, food animals, surveillance, epidemiology, bacteria, Europe, food safety

## Abstract

In Europe, systematic national surveillance of antimicrobial resistance (AMR) in food-producing animals has been conducted for decades; however, geographic distribution within countries remains unknown. To determine distribution within Europe, we combined 33,802 country-level AMR prevalence estimates with 2,849 local AMR prevalence estimates from 209 point prevalence surveys across 31 countries. We produced geospatial models of AMR prevalence in *Escherichia coli,* nontyphoidal *Salmonella*, and *Campylobacter* for cattle, pigs, and poultry. We summarized AMR trends by using the proportion of tested antimicrobial compounds with resistance >50% and generated predictive maps at 10 × 10 km resolution that disaggregated AMR prevalence. For *E. coli,* predicted prevalence rates were highest in southern Romania and southern/eastern Italy; for *Salmonella,* southern Hungary and central Poland; and for *Campylobacter*, throughout Spain. Our findings suggest that AMR distribution is heterogeneous within countries and that surveillance data from below the country level could help with prioritizing resources to reduce AMR.

Antimicrobial resistance (AMR) is a substantial threat to the health of humans and animals. Among humans, in 2019 an estimated 1.27 million deaths were associated with bacterial AMR (*1*). Among food-producing animals (i.e., animals that are used for or produce food items for human consumption), estimates of global AMR burden are still lacking. However, recent work has suggested that among common indicator bacteria of food-producing animals in low- and middle-income countries, the proportion of antimicrobials with resistance >50% increased from 12%–15% in 2000 to 34%–41% in 2018 (*2*), an increase that may have harmful consequences for humans (*3*). Moreover, the loss of treatment effectiveness in animals is a long-term threat for animal production and the millions of persons who rely on raising animals for subsistence (*4*,*5*). Therefore, monitoring AMR in food-producing animals has become a global priority for effective prevention strategies.

Since 2009, the European Food Safety Authority (EFSA) has led a harmonized surveillance system for AMR in food-producing animals and products (*6*). The system includes AMR prevalence estimates for *Escherichia coli*, nontyphoidal *Salmonella*, and *Campylobacter* among cattle and pigs (odd years) and chickens and turkeys (even years) (*7*). Data collected by EFSA have been instrumental for monitoring AMR and for guiding policy decisions in the European Union (e.g., the 2018 ban on prophylactic use of antimicrobials in animals [*8*]). The efforts to document AMR have also enabled comparison between countries in Europe by estimating prevalence of AMR at the national level. However, recent works have shown that resistance levels in humans and animals can vary at a fine spatial scale, and accumulation of resistance genes in those areas may create geographic hotspots for AMR (*2*,*9*). Identifying geographic hotspots of AMR within countries could help with targeting interventions against AMR, such as improved farm biosecurity and targeted surveillance, where they might have the greatest benefits (*10*–*12*).

In that context, point prevalence surveys (PPSs) of AMR among food-producing animals, with data points collected at individual geographic locations, provide an opportunity to supplement the national estimates of AMR assembled by EFSA (*2*). The resulting mapped predictions could be used to help design regional antibiotic stewardship campaigns or target local investment in farm biosecurity (*12*). However, generating robust predictions of AMR pose at least 3 challenges. First, comparisons need to be made between the resistance trends inferred from PPSs and EFSA; second, subnational predictions should reflect resistance levels reported by EFSA at the national level; and third, an appropriate geospatial modeling approach must be developed to combine data collected at different spatial scales.

In this study, we disaggregated trends in AMR prevalence of *E. coli,* nontyphoidal *Salmonella*, and *Campylobacter* among cattle, pigs, and poultry. We used stacked geospatial models that supplement data from EFSA with individual PPSs to map predictions of AMR prevalence at a resolution of 10 × 10 km for 31 countries in Europe.

## Materials and Methods

### EFSA Data Collection

We reviewed annual EFSA reports published during 2011–2022 (*13*). We extracted country-level data on AMR prevalence (2009–2020), focusing on the percentage resistance to antimicrobials against *E. coli*, *Salmonella*, *Campylobacter coli*, and *Campylobacter jejuni*. We extracted information on country, year of isolation, animal type (cattle, pigs, chickens, turkeys), sample origin (slaughtered animal, living animal, or meat), bacteria, species, number of samples, antimicrobial tested, and resistance prevalence. We followed European Committee on Antimicrobial Susceptibility Testing (EUCAST) guidelines to assess microbiological resistance and used microdilution methods and epidemiologic cutoff (ECOFF) values (*14*). We retained only antimicrobial/bacteria combinations recommended by the World Health Organization Advisory Group on Integrated Surveillance of Antimicrobial Resistance (*15*) for antimicrobial susceptibility testing (Appendix Table 1).

### PPS Data Collection

We systematically reviewed PPSs (Appendix) reporting AMR prevalence at individual locations in Europe (Appendix Figure 1). We searched PubMed, Web of Science, and Scopus for PPSs reporting AMR prevalence for *E. coli*, nontyphoidal *Salmonella*, and *Campylobacter* in healthy cattle, pigs, and poultry (combined data for chickens, turkeys, or other poultry), as well as their products (meat and dairy) in Europe during 2000–2021. Environmental samples (e.g., water, soil) were not included. We also extracted information on the geographic location of the PPS (Appendix), the year the PPS was conducted, the year the bacteria was isolated (but not species identification methods used), sample types collected (cecal, cloacal, lymph, or fecal samples taken from living animals, slaughtered animals, dairy products, or meat), animal species, number of samples collected and tested, susceptibility testing guidelines used, and susceptibility guidelines used for resistance interpretation.

We assessed microbiological resistance across PPSs by using different methods (disk diffusion vs. broth dilution), guidelines (Clinical and Laboratory Standards Institute [https://www.clsi.org] 52%, EUCAST 29%, other 14.6%) and cutoffs (clinical break points vs. ECOFFs [*15*]). We attempted to account for these differences by using a harmonization approach developed by Van Boeckel et al. (*2*) (Appendix). We calibrated data from PPSs by using antimicrobial susceptibility testing, guidelines, and breakpoints reported in each study to match those of EUCAST guidelines each year, to enable comparison between those data and data reported by EFSA. As with EFSA data, we retained only antimicrobial/bacteria combinations recommended by the World Health Organization Advisory Group on Integrated Surveillance of Antimicrobial Resistance (*15*). In addition, for our analysis we retained only countries that reported to EFSA and that had reported >50 samples during the study period. All prevalence estimates extracted from PPS are available at resistancebank.org (https://resistancebank.org) (*16*).

### Comparative Analysis of Data Sources

We used the proportion of antimicrobials with >50% resistance (P50s) to summarize trends in resistance across each drug/bacteria combination, as in previous works (*2*,*12*,*17*); all P50s can be recalculated by using the data available at resistancebank.org. To assess the difference in AMR prevalence between PPS and EFSA data, as well as the implications that that could have for geospatial modeling, we compared the average P50 in countries reporting at >1 PPS and to EFSA during 2018–2020 (Appendix Table 3). A ratio <1 indicated a lower 3-year mean P50 using PPS data, and a ratio >2 meant a more than double 3-year mean P50 from PPS data compared with EFSA data.

### Geospatial Modeling of P50

We mapped predicted subnational antimicrobial resistance in food-producing animals at a resolution of 0.08333 decimal degrees, corresponding to ≈10 km at the equator. To create the map, we used a 3-step procedure (Appendix Figure 2).

In the first step, we trained 3 child models (one of the individual models that are combined to form the final model) to quantify the relationship between P50 and a set of 9 environmental and anthropogenic covariates (Appendix Table 2). We selected those covariates because of their suspected association with AMR in animals (*2*,*12*,*17*–*19*). The models used for the first step were boosted regression trees (*20*); LASSO (least absolute shrinkage and selection operator) applied to logistic regression (*21*); and overlapped grouped LASSO penalties for General Additive Models selection (A. Chouldechova, unpub. data, https://arxiv.org/abs/1506.03850). We calculated the importance of each covariate by comparing the areas under the receiver operator curve (AUCs) between a full model that contained all covariates and a model without each covariate. To evaluate the relative importance of each covariate to the full model, we repeated the procedure sequentially (Appendix Table 5).

**Table 2:** Percentages of food-producing animals raised in each country that fall within an antimicrobial resistance hotspot area (95th percentile per pathogen) for France, Germany, Spain, and countries in which pathogen percentage >50% for >1 animal species*

Pathogen, country	Cattle, %	Pigs, %	Poultry, %
*Escherichia coli*			
France	0	0	0
Germany	0	0	0
Spain	2.1	2.3	1.8
Bulgaria	34.4	51.5	57.8
Cyprus	33.8	68.9	68.5
Greece	39.4	57.9	35.5
Romania	34.8	77.5	57.8
*Salmonella*			
France	0	0	0
Germany	0	0	0
Spain	8.8	28.2	24.8
Cyprus	51.8	91.6	96.6
Hungary	63.5	64.7	80.6
Italy	52.0	70.2	64.0
Poland	21.6	66.0	74.3
Romania	17.0	65.2	45.0
*Campylobacter*			
France	0.5	4.6	6.2
Germany	1.8	14.9	23.2
Spain	32.3	87.9	93.0
Cyprus	26.0	44.9	66.3
Greece	10.9	58.4	90.3
Portugal	22.1	74.9	88.0

*Antimicrobial resistance for *Escherichia coli*, nontyphoidal *Salmonella*, and *Campylobacter*.

We weighted all models by the number of isolates tested in each survey and conducted 10 Monto Carlo simulations on the models to account for the variation introduced by transformation of prevalence estimates into binary variables. The models were trained by using 4-fold spatial cross-validation to prevent overfitting and ensure generalization in geographic regions poorly represented in the training dataset. We defined the 4 spatial folds by using a k-means clustering algorithm (*22*). The algorithm clustered the surveys according to their spatial distances and partitioned them into 4 spatially disjointed sets with equal sizes (Appendix). No predictions were made in urban settlements; there were areas defined as artificial surfaces in GlobCover 2009 (*23*). We conducted sensitivity analyses by restricting PPSs to 2009–2020 only (to match EFSA reporting period), to 6 or 7 of the most common antimicrobial/bacteria combinations only, and to P50 calculated by class (rather than compound) (Appendix).

In the second step, we ensembled predictions from the 3 models according to the models’ predictive ability, assessed by using the AUC. We calculated the resulting map of P50 as the mean of the 3 model predictions weighted by their AUC values. We calculated the associated map of prediction uncertainty as the SD of predicted P50 values from the 10 Monte Carlo simulations (Appendix Figure 4, panel A).

In the third step, we adjusted the P50 predictions in each country, using P50 values calculated from EFSA reports. Concretely, we multiplied P50 values in each pixel by the ratio of country-level P50 as reported by EFSA and the mean P50 of all pixels across each country as predicted by the geospatial model. That step ensured that the country-level mean of P50 values corresponded to reports from EFSA while preserving geographic variations in AMR levels within each country. To assess the variations in P50 values within each country, we calculated country-level SDs of P50s (Appendix Figure 4, panel C).

Last, we created the predictive maps of AMR hotspots for each pathogen. The threshold value for a pixel to be classified as a hotspot corresponded to the 95th percentile of all P50 values across the map and varied for each pathogen (Appendix Figure 4, panel B). We obtained estimated animal densities associated with those areas from Gilbert et al. (*24*). Using those estimates, for each country we calculated the percentage of each animal species living in the hotspot areas.

## Results

### EFSA Surveillance

At the country level, EFSA data for 2009–2020 provided 33,802 AMR prevalence estimates (resulting in 2,996 P50s). The data were for *E. coli*, nontyphoidal *Salmonella*, *C*. *coli*, and *C. jejuni* in cattle, pigs, and poultry across 31 countries in Europe.

### PPSs

**Figure 1:**
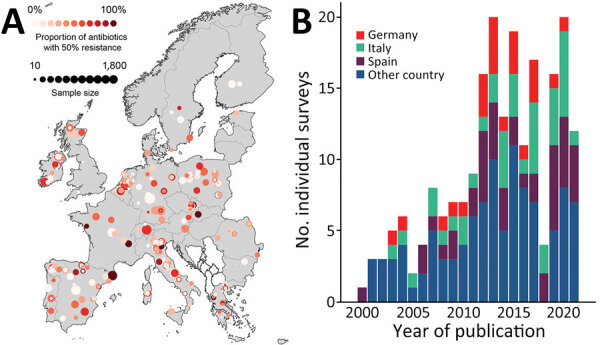
Data from study of predictive mapping for antimicrobial resistance of *Escherichia coli*, *Salmonella*, and *Campylobacter* in food-producing animals, Europe, 2000–2021. A) Geographic distribution of point prevalence surveys (PPSs). B) Number of PPSs published per year. Additional information is provided in the Appendix.

At the local level, for 2000–2021 we identified 209 PPSs, which provided 2,849 AMR prevalence estimates (resulting in 368 P50s). The data were for *E. coli*, nontyphoidal *Salmonella*, and *Campylobacter* in food-producing animals and derived products from 21 countries in Europe. In terms of AMR prevalence, *E. coli* accounted for 44.4%*,* *Salmonella* for 34.2%, and *Campylobacter* for 21.4%*.* Poultry accounted for approximately half of the AMR prevalence (n = 1,429, 50.2%), followed by pigs (28.1%) and cattle (21.8%). One third of the sample types tested were meat (34.7%, n = 988), followed by fecal samples (23.4%). Across the countries included in the analysis, geographic coverage was on average 4.21 PPSs (interquartile range 0–11.7)/100,000 km^2^. Half of the PPSs identified were from the combination of Spain (20.5%), Italy (18.7%), and Germany (10.5%) (Figure 1). The average number of PPSs published by year increased from 3 during 2000–2005 to 14 during 2015–2021 (Figure 1, panel B).

### Comparison of PPS and EFSA

AMR prevalence estimates varied considerably between data sources and country. For 2018–2020, Greece, Poland, and Germany accounted for more than double the national average P50 calculated from PPS data compared with P50s calculated from EFSA (Table 1). Conversely, the national average P50 calculated from PPS data from Portugal and Switzerland was <30% lower than that calculated from EFSA.

**Table 1:** Three-year mean of proportion of antimicrobial drugs with >50% resistance from PPS and EFSA data and ratios of P50 for countries reporting to both data sources, Europe, 2018–2020*

Country	Mean P50 from PPSs	Mean P50 from EFSA	PPS and EFSA P50 ratio
Poland	0.64	0.26	2.47
Germany	0.60	0.25	2.42
Greece	0.39	0.19	2.02
Spain	0.39	0.24	1.67
Belgium	0.29	0.21	1.34
Romania	0.31	0.28	1.10
Italy	0.23	0.25	0.92
Switzerland	0.17	0.22	0.77
Portugal	0.18	0.32	0.57

*EFSA, European Food Safety Authority; PPS, point prevalence surveys; P50, >50% antimicrobial resistance.

**Figure 2:**
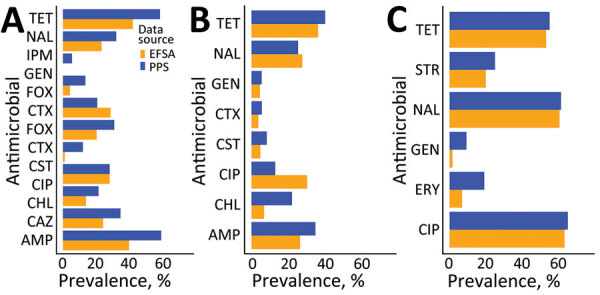
Mean prevalence for antimicrobial class and bacteria combinations, split by data source, Europe, 2009–2020. A) *Escherichia coli*; B) *Salmonella*; C) *Campylobacter*. AMP, ampicillin; CAZ, ceftazidime; CHL, chloramphenicol; CIP, ciprofloxacin; CST, colistin; CTX, clavulanic acid; EFAS, European Food Safety Authority; FOX, cefoxitin; GEN, gentamicin; IPM, imipenem; NAL, nalidixic acid; PPS, point prevalence survey; STR, streptomycin; TET, tetracycline.

The highest resistance prevalence estimates were for tetracycline (57.9%–36.4%), ampicillin (58.6%–34.9%), ciprofloxacin (64.6%–13.1%), and nalidixic acid (60.9%–25.5%). The difference in mean P50 between PPSs and EFSA data ranged from 15.2% to −17.4% for *Salmonella* and from 19.1% to −7.96% for *E. coli.* For *Campylobacter*, systematically higher prevalence estimates were obtained from PPSs; differences ranged from 12.1% to 0.78% (Figure 2).

### Geospatial Modeling

**Figure 3:**
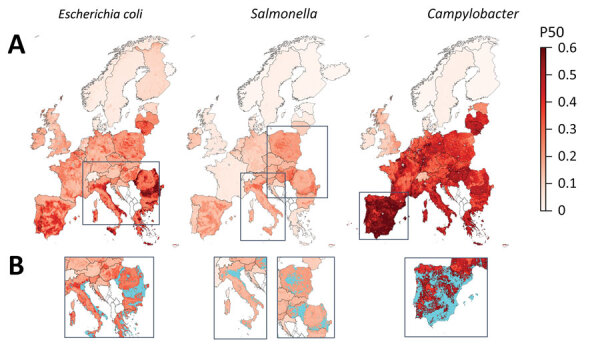
Mapping of predicted P50s and hotspot areas for antimicrobial resistance of *Escherichia coli*, *Salmonella*, and *Campylobacter*, Europe. A) Predicted proportions of antimicrobials with P50 at 10 × 10 km resolution per bacteria. B) Antimicrobial resistance hotspots (light blue) in eastern Europe, Italy, and Spain. Cutoffs: *E. coli*, 0.43; *Salmonella*, 0.23; *Campylobacter*, 0.6 (95% percentile). P50, >50% antimicrobial resistance.

We mapped predicted P50s at 10 × 10 km resolution for each of the 3 bacteria across Europe (Figure 3). In the final models, the predicted P50 values ranged from 0 to 79% for *E. coli,* 0 to 40% for *Salmonella*, and 0 to 100% for *Campylobacter* (Figure 3, panel A; prediction uncertainty, Appendix Figure 3, panel A). P50 cutoffs for hotspots of AMR (calculated as the top 95% of the values on the map) were 0.43 for *E. coli*, 0.23 for *Salmonella*, and 0.60 for *Campylobacter*. AMR hotspots for *E. coli* were predicted to be located in southern Romania (Muntenia, Dobrogea) and southern and eastern Italy (Sicily, Emilia-Romagna, Apulia); and for *Salmonella*, predicted hotspots were in southern Hungary, northern Italy, and central Poland. More than 90% of hotspot areas for *Campylobacter* were predicted to be throughout mainland Spain (Figure 3, panel B).

For *E. coli*, the highest geographic variations in predicted P50 levels were in Romania (13% pixel-level SDs), Bulgaria (11%), Greece 1(2%), and Italy (11%). For *Campylobacter*, the highest geographic variations in P50 were in France (10%) and Germany (10%; Appendix Figure 4, panel C). No countries had high spatial variations in predicted P50s for *Salmonella*. Cold spots for all 3 bacteria were identified in Sweden, Norway, Finland, and Iceland (data not shown). Spatial variations of P50 for countries containing coldspots were small, with pixel-level standard deviations of 3.2% (*E. coli*), 0.9% (*Salmonella*), and 1.0% (*Campylobacter*). Restricting PPS by year and antimicrobial bacteria combinations resulted in little difference (mean Pearson correlation coefficient 0.992; mean absolute error 0.932%) to the overall model predictions (Appendix Table 4). In addition, we found little difference when P50 was calculated by antimicrobial class rather than individual compound (Pearson correlation coefficient 0.995, mean absolute error 0.66%) (Appendix Table 4, Figure 4). Importance of environmental covariates to the models varied by pathogen (Appendix Table 5). For *E. coli* and *Salmonella*, the covariate with highest importance was the percentage of tree coverage (∆AUC 0.106 for *E. coli* and 0.078 for *Salmonella*). For *Campylobacter*, the covariate with highest importance was antimicrobial use in animals (∆AUC 0.037), closely followed by yearly average of minimum monthly temperature (∆AUC 0.034).

In 9 of the 31 countries in Europe, >50% of cattle, pigs, or poultry are estimated to be raised in the predicted AMR hotspot areas (Table 2). For instance, 93% of poultry in Spain, 90% of poultry in Greece, and 97% of poultry and 92% of pigs in Cyprus are raised in AMR hotspots.

## Discussion

In this study, we geographically disaggregated AMR prevalence for *E. coli*, nontyphoidal *Salmonella*, and *Campylobacter* reported among food-producing animals across Europe by supplementing national EFSA data with subnational PPS data to produce maps of estimated AMR prevalence. For multiple countries, such as Italy, Romania, and Poland, rather than consistently high countrywide AMR levels, in our final model we predicted specific geographic hotspots of high AMR prevalence that may coexist within regions of lower AMR prevalence in the same countries. In specific regions, countries in which AMR seems to be consistently high may have made more progress against AMR than previously thought (with only some, rather than all, areas containing high levels) by interpretation of EFSA data or nationally published reports. Further improvements could be made in those countries by targeting interventions (e.g., improved farm biosecurity and targeted surveillance in hotspots where AMR levels remain high). In contrast, largely diffuse and geographically uniform (low) countrywide AMR prevalence was found in countries with low AMR levels (e.g., Sweden, Norway, and Iceland); uncertainty in these predictions were higher for *Campylobacter* than for *E. coli* and *Salmonella*.

For all 3 bacteria studied, AMR prevalence was substantially lower in Norway, Sweden, Denmark, and Switzerland than the average for Europe. Those countries were among the first to establish animal AMR surveillance (i.e., DANMAP in Denmark in 1995 [*25*]) and have now integrated surveillance of zoonotic bacteria in humans and animals. For several decades, they have been guiding national and international control strategies. For instance, in the 1990s, increased prevalence of vancomycin-resistant enterococci reported by DANMAP was instrumental to banning use of antimicrobial drugs for growth promotion in livestock (*25*).

In contrast, countries in which a high proportion of food-producing animals are raised in areas predicted as hotspots of resistance by our study are Cyprus, Portugal, and Spain. In 2018, one fifth (20.8%) of the pigs in the European Union were reared in Spain (*26*), where 88% of its pigs were predicted to be raised in geographic hotspots of *Campylobacter* resistance, primarily in Aragon and Catalonia. However, that finding was not the case for other high-density pig regions such as Brittany (France), northwest Germany (Lower Saxony and North Rhine-Westphalia), and Denmark (*27*). Those findings suggest that high AMR is not necessarily associated with high animal densities but possibly with other drivers such as farming practices, biosecurity measures, and antimicrobial use (*28*).

Across Europe, the highest prevalence of resistance in our models was reported for antimicrobial drugs commonly used in animal production: tetracyclines, quinolones, penicillins, and aminoglycosides (gentamicin and streptomycin). Of particular concern were the compounds considered critically important antimicrobials for human medicine (*29*) and for which AMR prevalence was predicted to be >50% (ampicillin in *E. coli* [58.6%] and ciprofloxacin in *Campylobacter* [64.6%]).

In our study, estimates of P50 for *Salmonella* were much lower than those for *E. coli* and *Campylobacter*, which could potentially be attributed to the success of targets imposed by the European Union (e.g., reducing *Salmonella* prevalence in poultry over the past decade [*30*]). In addition, several countries had already implemented *Salmonella* control strategies before European Union–wide initiatives. For instance, in the 1970s, the United Kingdom set up national AMR surveillance for *Salmonella*, and in 1969, France had similar initiatives for *Salmonella* and *E. coli* (*25*). Switzerland also implemented a stringent control program for *Salmonella* Enteritidis in 1993 (*31*), more than a decade earlier than the first European Union–wide initiative (*30*).

When we compared estimates of resistance (P50) derived from PPS and EFSA data, the average P50 from PPSs seemed to more closely match national EFSA prevalence values in some countries more than in others. For instance, in Spain and Italy, the ratios of P50 inferred from PPS and EFSA data were close to 1 over the past 3 years. One reason may be the higher number of PPSs from these countries (17 in Spain and 13 in Italy), which average out closer to the EFSA values. In contrast, in countries with P50 ratios >2 or <0.8 (Poland, Germany, Greece, Portugal) inferred from PPS and EFSA data, only 1–4 studies have been conducted in the past 3 years. Therefore, although smaller sample sizes may be insufficient for comparing national averages (PPS vs. EFSA) they may still represent subnational heterogeneity in AMR not observed in the national average from EFSA. A higher coverage of PPSs may further improve the confidence in subnational model predictions.

Among the limitations of our modeling study, the first is that our literature search for PPSs published in Europe during 2000–2021 resulted in a mere 209 PPSs that were associated with geographic information. In contrast, for the same period, 446 PPSs with geographic information were published in China (*12*). Torres et al. also assembled AMR studies of food-producing animals during 1957–2018; however, of the 510 papers from Europe identified, the breakdown of their surveys corresponding to our search criteria was not available in open access (*32*). Thus, the limited number of surveys that satisfied our inclusion criteria, particularly the reporting of geographic information, precluded mapping AMR prevalence for individual drug/bacteria combinations or animal species.

Second, with regard to using PPSs for regional estimations, differences in sampling strategy and sample sizes may affect the comparability of surveys and potentially explain why prevalence calculated from PPSs was in some instances higher than the prevalence estimates reported by EFSA. In particular, targeted sampling for bacteria that probably have high-resistance profiles, such as extended-spectrum beta lactamase–producing *E. coli* (*33*), could lead to comparatively higher AMR in PPS data than in the general population, which are more likely to be observed with the EFSA sampling scheme. In terms of microbiology, the set of tested antimicrobials differed between PPSs, which necessitated use of a composite metric. In addition, there were some transparency issues in terms of which methods or breakpoints were used (i.e., assumptions had to be made in the case of missing data [such as guideline year] and in the harmonization approach used for PPSs that used different guidelines, which may have led to some unintended bias), as well as a diversity of breakpoints used. Despite attempts to reduce variability between surveys, some variability may still exist and therefore efforts should be made to develop standardized protocols in the future, such as for all PPSs to shift to using ECOFF values and to release raw data. The creation of a consensus breakpoint table that could be used by all would also greatly assist with the comparability of those data and reduce the need for such adjustments. Because most studies reported only sampling location or region by name rather than specific coordinates, coordinates and size of region were estimated (and may not always represent the location of the farms where the animals were raised), which may have led to further uncertainty in our models.

Third, because of the limited number of PPSs, as well as their heterogenous distribution across the study period, incorporating the temporal dimension into the modeling framework remains challenging at this stage. Therefore, countries that have had considerably reduced AMR levels since 2009, such as the Netherlands (*34*), may be associated with higher AMR prevalence in our maps than that in the latest reports. However, as the number of surveys grows in the future, other spatio-temporal approaches, such as the Integrated Nested Laplace Approximation (*35*), could be used to account for not only spatial but also temporal variations in AMR prevalence extracted from PPSs.

Last, because of the static framework of geospatial modeling, it was not possible to incorporate all relevant data. That limitation may have a dynamic effect on AMR prevalence estimates, notably animal movement.

In conclusion, high-resolution maps that predict subnational hotspots can help support targeted resource allocation and control strategies for reducing AMR burden. Such strategies could include improving farm biosecurity and targeted surveillance. The accuracy of these maps could be gradually improved in the future should countries routinely report geographic location data along with microbiological sampling results.

Appendix Additional information for study of predictive mapping of antimicrobial resistance of *Escherichia coli*, *Salmonella*, and *Campylobacter* in food-producing animals, Europe, 2000–2021.
