# Supplementary material for: Predictive Mapping of Antimicrobial Resistance for Escherichia coli, Salmonella, and Campylobacter in Food-Producing Animals, Europe, 2000–2021
Source: Emerg Infect Dis. 2024 Jan;30(1):96–104. doi: 10.3201/eid3001.221450 (PMC10756390; doi:10.3201/eid3001.221450)
Supplement: Appendix — Additional information for study of predictive mapping of antimicrobial resistance of Escherichia coli, Salmonella, and Campylobacter in food-producing animals, Europe, 2000–2021. [file 22-1450-Techapp-s1.pdf]

Article DOI: https://doi.org/10.3201/eid3001.221450

EID cannot ensure accessibility for supplementary materials supplied by authors. Readers who have difficulty accessing supplementary content should contact the authors for assistance.

Predictive Mapping of Antimicrobial Resistance for *Escherichia coli*, *Salmonella,* and *Campylobacter* in Food-Producing Animals, Europe, 2000–2021

Appendix

Methods

Literature Review

We conducted a systematic literature review on antimicrobial resistance (AMR) prevalence in livestock and livestock products in Europe (Appendix Figure 1). We used three databases: PubMed, ISI Web of Science, and Scopus. Our original search focused on four pathogens commonly found in animals and their products*: Escherichia coli*, *Staphylococcus aureus, Campylobacter* spp., and non-typhoidal *Salmonella* spp. The searches were conducted at different time periods between May 2019 and January 2022, and included studies published between 2000 and 2021.

The general format for our literature queries was: (Resistance) AND (Bacterial Species) AND (Animal/Sample Type) AND (Country).

The keywords used for the literature review on PubMed, ISI Web of Science, and Scopus were: ("antibiotic resistance" OR "antimicrobial resistance" OR resistance OR resistencia OR "resistencia aos antibioticos" OR resistencia OR "resistencia a antibioticos" OR susceptibility OR susceptibilidade OR suscetibilidade OR antibiogram OR "antibiotic susceptibility testing" OR antibacteriano OR antibiotic OR antimicrobial OR antibiotic OR antibacterial OR antimicrobiano OR antibiograma OR antibiotic) AND (Escherichia OR "E. coli" OR coliform OR salmonella OR "salmonella spp." OR "S. aureus" OR staphylococcus OR "Staphylococcus spp." OR "MRSA" OR "MSSA" OR campylobacter OR "campylobacter spp." OR "C. jejuni" OR "C.

coli") AND (animal OR food OR "food producing" OR meat OR cow OR cattle OR beef OR bovine OR buffalo OR pig OR piggeries OR pork OR "chicken" OR "flock" OR "broiler" OR "layer" OR "egg" OR "poultry" OR "avian" OR milk OR dairy OR cheese) AND (France OR Spain OR Netherlands OR Denmark OR Sweden OR Italy OR Greece OR Germany OR French OR Spanish OR Dutch OR Danish OR Swedish OR Italian OR Greek OR German OR Norway OR Norwegian OR Finland OR Finnish OR Poland OR Polish OR "United Kingdom" OR England OR English OR Romania OR Romanian OR Bulgaria OR Bulgarian OR Iceland OR Icelandic OR Hungary or Hungarian OR Portugal OR Portuguese OR Austria OR Austrian OR Czechia OR "Czech Republic" OR Czechian OR Ireland OR Irish OR Lithuania OR Lithuanian OR Latvia OR Latvian OR Croatia OR Croatian OR Slovakia OR Slovakian OR Estonia OR Estonian OR Switzerland OR Swiss OR Moldova OR Moldovan OR Belgium OR Belgian OR "North Macedonia" OR Macedonia OR Macedonian OR Slovenia OR Slovenian OR Cyprus OR Luxembourg OR Malta OR Maltese). In PubMed, this query was put directly into the search bar. On Scopus, this search was conducted using TS = (keywords given above), where TS stands for our search topic. In the ISI Web of Science, the search was conducted using TITLE-ABS-KEY = (keywords given above). Here, TITLE-ABS-KEY stands for title, abstract, and keywords.

In PubMed, Scopus, and ISI Web of Science, an initial search on eight European countries (Italy, Germany, the Netherlands, Spain, France, Greece, Denmark, and Sweden) was conducted on January 7, 2020, for Point Prevalence Survey (PPS) published between 2000 and 2019. These searches yielded 14,445 results. Titles and abstracts were screened manually. After removing duplications, reviews, meta-analyses, book chapters, and papers irrelevant to our topic of interest, we had 1,265 potentially relevant manuscripts. At this point, papers were read and removed if geographic data was unavailable, no antimicrobial susceptibility testing was performed, the study focused on sick animals, the survey focused on animals at the country-wide level, results were pooled between different animal species or sample types, or resistance prevalences were pooled between different pathogen types. From these, 191 papers were extracted, yielding 4853 resistance estimates.

Next, in PubMed, Scopus, and ISI Web of Science, a search for the remaining European countries was conducted on April 23, 2020, for PPS published between 2000 and 2019. This search yielded 54,591 results. Titles and abstracts were screened in the same manner as the first

search, and the same non-relevant results were removed. After this step, we had 745 potentially relevant manuscripts. From these, 98 were extracted, yielding 1567 resistance prevalences.

In PubMed, Scopus, and ISI Web of Science, a search for all European countries was conducted on January 7, 2021, for PPS published in 2020. This search yielded 6,005 results. Titles and abstracts were reviewed in the same manner as the previous two searches, and the same non-relevant results were removed. After this step, we had 253 potentially relevant manuscripts. From these, 34 were extracted, yielding 783 resistance prevalences.

A final literature search (identical to that of January 7, 2021) was run on January 10, 2022, to identify all PPS published in 2021. This search yielded an additional 6,598 results. As outlined previously, all the same steps for title and abstract screening were followed, leaving 110 potentially relevant manuscripts. Of these, 22 were extracted, yielding 606 resistance prevalences. Overall, this gave 345 papers with 7,809 resistance estimates of any antibiotic-pathogen combination.

As there was no mandated or routine reporting of *Staphylococcus aureus* to EFSA (there was only limited voluntary reporting of MRSA from 5 countries in 2018 and 6 countries in 2019) AMR estimates for *S. aureus* were subsequently excluded. Additionally, only countries reporting to EFSA were retained. The final number of manuscripts was 209.

Geographic localization of point prevalence surveys

Only PPS that reported geographic information were included in the study. The extracted information was recorded in "name_of_location" and "level_of_uncertainty" variables.

- "Name_of_location" contains the name(s) of the most precise location information available in the article. Where more than one location was reported, both names were recorded.
- "Level_of_uncertainty" contains the administrative level at which the sampling was performed (see Legend on resistancebank.org for full details). These data were then used to determine "Ycoord" and "Xcoord" variables.
- These data were then used to determine "Ycoord" and "Xcoord" variables. There were two ways in which these were generated:

1. **Samples taken from across an area/province** – the centroid of the province was obtained.
2. **Several sampling points across an area/region** – the middle point of all the sampling points was taken. This can be identified using variable "name_of_location" where more than one name is recorded.

Example:

DOI: 10.1155/2009/456573

Extract 1 from paper: "C. jejuni isolates were selected from a prevalence study of thermophilic campylobacters in livestock carried out in the Basque Country (Northern Spain)"

Extract 2 from paper: "…isolates were selected on the basis of isolation source (host, farm, and flock). Hence, the 72 isolates analysed by broth microdilution included 19 isolates from 12 poultry farms (18 flocks), 25 from dairy sheep (21 farms), and 28 isolates from cattle (14 beef cattle and 11 dairy cattle farms)"

Interpretation: Tested a specific subset of isolates from across Basque.

Level of uncertainty: Province

Name of location: Basque County

X/Y Coordinates: taken from the centroid of the Basque province.

Harmonization of antimicrobial resistance rates

The two most frequently used systems for antimicrobial susceptibility testing (AST) are Clinical and Laboratory Standards Institute (CLSI) and European Committee for Antimicrobial Susceptibility Testing (EUCAST). Each system uses breakpoints to classify susceptible and resistant phenotypes; these values are updated annually. Therefore, adjustment for breakpoint variation over time is essential.

In this study, we found 96% of records reported the guidelines used, while 72% of these records also reported the year of the guidelines used. The majority of records reported CLSI (52%), followed by EUCAST (29%), despite all studies originating from the European region. 4.4% of records did not report a guideline, and these records were excluded from subsequent analysis. The remaining records reported a mix of guidelines used in mentioned surveillance

systems (e.g., DANMAP, NARMS, BSAC etc). For records where the guideline was reported, but no specific year, a date 4 years prior was assumed as this was the median lag between publication date of the survey and year of the guidelines. These assumptions were applied to maximise the amount of data retained for subsequent analyses.

The same harmonization procedure was then applied to all records as outlined in reference (*1*). This harmonization procedure resulted in 9% of records (262 out of 2888) being revised.

To assess the impact of using CBPs rather than ECOFFs, we changed the breakpoints used to ECOFFs rather than CBP, which resulted in 11% of the calculated P50s changing. Of these 38 P50s, the average absolute change was 18.9%. For these P50s, the majority (n = 35) became larger, while five became smaller. Therefore, ≈90% of the calculated P50s would remain the same if the breakpoints were changed, and the absolute change would be relatively small.

Desk review of national reports

We conducted a desk review of European countries to identify national reports that contain information on AMR in food-producing animals (Appendix Table 6). The contents of the reports were compared with EFSA, to determine if there was any further relevant data contained within these reports. Due to the limited additional data, with low comparability, these data were not extracted for this study.

Geospatial modeling of P50

During the first step, the P50 values (proportions) were transformed into presence and absence of resistance using a random binarization procedure. Concretely, each P50 value was duplicated 5 times, and compared with a random number between 0 and 1. P50 values higher than the random number were classified as presence of resistance, otherwise the values were classified as absence of resistance.

Sensitivity analyses and covariate importance

Sensitivity analyses were conducted by (a) restricting PPS to the same period as EFSA (2009–2020), (b) restricting to the six/seven most common drug-bug combinations and (c) by calculating P50 by class of drug rather than individual compound. For analysis (b), for *E. coli* and *Salmonella* the seven drugs included were: TET, AMP, SXT, CHL, CIP, GEN, CTX. The six most common drugs for *Campylobacter* were AMP, STR, GEN, CIP, TET, ERY.

The importance of covariates was calculated by sequentially removing each covariate from the modeling procedure and comparing the changes in the mean AUC across 10 Monte Carlo simulations.

Results

Descriptive analysis

A total of 81,639 records were identified from the literature search (Appendix Figure 1). Following de-duplication, title, abstract and subsequent full paper screen, a total of 209 studies with geographic information had data extracted. From 209 PPS where geographic information was reported, 2,849 AMR estimates were extracted, providing 368 P50s.

From the EFSA reports, 2,996 P50s at country-level (33,802 AMR estimates) were calculated from data collected between 2009 and 2020. The numbers of countries reporting to EFSA each year ranged from 23 in 2009, 20 in 2011, to 31 countries reporting annually from 2015 onwards.

Appendix Table 1. Suggested antimicrobials, by bacteria, for inclusion for antimicrobial susceptibility testing (AST) for surveillance of AMR in foodborne bacteria (*2*)

Antimicrobial classes	*Salmonella, E. coli*	*Campylobacter*
Aminoglycosides	Gentamicin	Gentamicin<br>Streptomycin
Amphenicols	Chloramphenicol	
Carbapenems	Imipenem<br>Meropenem	
Cephalosporins II	Cefoxitin	
Cephalosporins III	Cefatoxime (or Ceftriaxone)<br>Ceftazidime	
Cephalosporins IV	Cefepime	
Glycopeptides		
Glycylcyclines	*Tigecycline*	
Lincosamides		*Clindamycin*
Lipopeptides		
Macrolides	Azithromycin	Erythromycin*
Nitrofurans	*Nitrofurantoin*	
Oxaxolidinones		
Penicillins	Ampicillin<br>Amoxicillin<br>*Temocillin*	Ampicillin
Polymyxins	Colistin	
Quinolones	Ciprofloxacin<br>Nalidixic acid<br>*Pefloxacin*^	Ciprofloxacin<br>*Nalidixic acid*
Rifamycins		
Streptogramins		
Sulfonamides	Sulfisoxazole#	
Tetracyclines	Tetracycline	Tetracycline˜
Trimethoprim	Trimethoprim	

Antimicrobials italicized are second priority
* Resistance toward erythromycin reflects azithromycin resistance
^ To screen for ciprofloxacin resistance in *Salmonella* spp. when disk diffusion is used.
# Trimethoprim-sulfamethoxazole can be used instead of using sulfisoxazole or trimethoprim alone
˜ Doxycycline may be used instead of tetracycline

Appendix Table 2. Environmental and anthropogenic covariates use to train child models

Name	Acronym	Year	Original Resolution	Source	Unit
Travel time to cities	acc	2015	30-arcsec resolution	(*3*) https://www.map.ox.ac.uk/accessibility_to_cities/.	minute
Antimicrobial use in animals	use	2013	0.083333 decimal degrees	(*4*) http://science.sciencemag.org/content/357/6358/1350.full	Log10[(mg/pixel)+1]
Yearly average of minimum monthly temperature	tmp	1970–2000	2.5 min	(*5*) http://worldclim.org/version2	°C * 10
Percentage irrigated areas	irg	2015	0.083333 decimal degrees	Global Map of Irrigation Areas (GMIA) (6) https://zenodo.org/record/6886564#.YuZ1HS8RpN0	%
Population density of cattle, chickens, pigs, and sheep (GLW version 4)	ca_v4 ch_v4 pg_v4 sh_v4	2015	0.083333 decimal degrees	(*7*) (https://www.nature.com/articles/sdata2018227)	Log10[(Heads/pixel) +1]
Percentage of tree coverage	veg	2013	0.008333 decimal degrees	(*8*) https://earthenginepartners.appspot.com/science-2013-global-forest/download_v1.2.html	%

Appendix Table 3. Absolute difference between resistance prevalence for antimicrobials by data source (point prevalence survey (PPS) vs European Food Safety Authority (EFSA)) between 2018 and 2020, and their WHO designation of antimicrobial importance*.

Variable	*E. coli*	*Salmonella*	*Campylobacter*	WHO Grouping
AMP	19.11	8.27	-	Critically important
CAZ	10.41	-	-	Critically important
CHL	7.51	15.2	-	Highly important
CIP	−0.02	−17.4	1.71	Critically important
CST	10.66	3.41	-	Critically important
CTX	10.41	1.87	-	Critically important
ERY	-	-	12.1	Critically important
FOX	−7.96	-	-	Highly important
GEN	9.09	0.96	7.66	Critically important
IPM	5.53	-	-	Critically important
NAL	8.7	−2.35	0.78	Critically important
STR	-	-	4.99	Critically important
TET	16.07	3.81	1.87	Highly important

*A ratio <1 indicated a lower 3-y mean P50 using PPS data, and a ratio >2 meant a more than double 3-y mean P50 from PPS data compared to EFSA.

Appendix Table 4. Comparison between maps produced using all extracted data, maps produced using restricted number of drugs, maps produced when P50 is calculated by class of drug (rather than individual compound), and maps produced using only surveys published between 2009 and 2020. Mae: mean absolute error; Cor: Pearson correlation coefficient.

Variable	*E. coli*	*Salmonella*	*Campylobacter*
(a) Restricted by year (2009–2020)			
No. of surveys	123	66	74
Mae	0.85%	0.75%	1.4%
Cor	0.994	0.986	0.995
(b) Restricted pathogen-antimicrobial combinations			
No. of surveys	153	97	111
Mae	1.5%	0.46%	0.63%
Cor	0.984	0.994	0.999
(c) P50 calculated at class level			
No. of surveys	156	99	113
Mae	1.0%	0.5%	0.49%
Cor	0.992	0.993	0.999

Appendix Table 5. Importance of covariates for mapping the distribution of AMR, indicating mean AUC of the full model, and the decrease in mean AUC after each covariate was removed from the modeling procedure.

Variable	*E. coli*	*Salmonella*	*Campylobacter*
Full model	0.635	0.606	0.536
Travel time to cities	0.03	0.001	0.02
Antimicrobial use in animals	0.019	0.037	0.037
Yearly average of minimum monthly temperature	0.033	0.042	0.034
Percentage irrigated areas	0.016	0.034	0.027
Population density of cattle	0.024	0.036	0.023
Population density of chicken	0.025	0.041	0.023
Population density of pigs	0.017	0.03	0.032
Population density of sheep	0.03	0.029	0.032
Percentage of tree coverage	0.106	0.078	0.024

Appendix Table 6. Desk review of European countries reporting AMR in zoonotic and foodborne bacteria (2007–2020)

No.	Country	EU Member State	Reporting to EFSA*	PPS extracted^	National level integrated surveillance (*9*)<br>Comparison of report content and frequency of data reporting compared to EFSA reports
1	Albania		✓		
2	Austria	✓	✓	✓	
3	Belgium	✓	✓	✓	
4	Bulgaria	✓	✓	✓	
5	Cyprus	✓	✓		
6	Czech Republic	✓	✓	✓	
7	Denmark	✓	✓	✓	✓ DANMAP (Last accessed 21 Feb 2022). Established 1995.<br>Pathogen & animal types:<br>• *Salmonella Typhimurium* (pig)<br>• *Salmonella Derby* (pig)<br>• *Campylobacter jejuni* (chicken, cow)<br>• *E. coli* (chicken, cattle)<br>Reporting content:<br>Same pathogens and animal types are reported to EFSA each year, with the same sample sizes.<br>Additional data available:<br>Last published report contains 2020 data.
8	Estonia	✓	✓	✓	
9	Finland	✓	✓	✓	✓ FINRES-VET (Last accessed 21 Feb 2022)<br>Pathogen & animal types:<br>• *Campylobacter jejuni* (chicken, cow)<br>• *Salmonella* spp. (pooled animal types)<br>Reporting content:<br>Report less in report than to EFSA.<br>Additional data available:<br>Last published report contains 2020 data.

No.	Country	EU Member State	Reporting to EFSA*	PPS extracted^	National level integrated surveillance (*9*) Comparison of report content and frequency of data reporting compared to EFSA reports
10	France	✓	✓	✓	✓ ONERBA (Last accessed 21 Feb 2022)<br>Pathogen & animal types<br>• *E. coli* (cattle, turkeys, pig)<br>Reporting content:<br>Only report mandatory data (e.g., in 2018 only reported on turkeys and chickens, and for the requested sample size). The ONERBA published report in 2018 contains larger samples sizes and contain additional data on pigs.<br>Additional data available:<br>Last published report only contains 2018 data, however historical reports contain additional animal types and larger sample sizes compared to EFSA.
11	Greece	✓	✓	✓	
12	Germany	✓	✓	✓	? GERMAP (no report publicly available since 2015)
13	Croatia	✓	✓		
14	Hungary	✓	✓	✓	
15	Iceland		✓		
16	Ireland	✓	✓	✓	
17	Italy	✓	✓	✓	
18	Lithuania	✓	✓		
19	Luxembourg	✓	✓		
20	Latvia	✓	✓		
21	Malta	✓	✓		
22	The Netherlands	✓	✓	✓	✓ MARAN (NETHMAP) (Last accessed 21 Feb 2022)<br>Pathogen & animal types:<br>• *Salmonella* spp. (pooled animal types)<br>• *E. coli* (pigs, chicken, cow, turkey)<br>Reporting content:<br>Only trends reported in prose. Data not in an extractable format.<br>Additional data available:<br>N/A – no extractable data available.
23	Norway		✓		✓NORM-VET (Last accessed 21 Feb 2022)<br>Pathogen & animal types<br>• *Salmonella* spp., but animals are pooled<br>• *Campylobacter jejuni* and *Campylobacter coli* (chicken, turkey, pigs)<br>• *E. coli* (chicken, turkey, cattle, pigs, goats)<br>Reporting content:<br>Animal types differ year-on-year, in-line with EFSA requirements; report same sample sizes.<br>E.g., in 2020, reported *E. coli* in chicken and turkeys while in 2019, reported *E.coli* in cattle and pig.<br>Additional data available:<br>Last published report contains 2020 data.
24	Poland	✓	✓	✓	
25	Portugal	✓	✓	✓	
-	Republic of North Macedonia		✓		*Excluded from geospatial analysis due to small numbers in EFSA data.*
26	Spain	✓	✓	✓	
27	Sweden	✓	✓	✓	✓ SVARM (Report - SWEDRES) (Last accessed 21 Feb 2022)<br>Pathogen & animal types:<br>• ESBL-producing *E.coli* (chicken) (no AST)<br>• *Salmonella* spp. (pooled animals)<br>• *Campylobacter jejuni* (chicken) and *coli* (pig)<br>Reporting content:<br>Report *Campylobacter* in-line with EFSA requirements.<br>Additional data available:<br>Last published report contains 2020 data.
28	Switzerland		✓	✓	ARC-Vet (Last accessed 21 April 2022)<br>Pathogen & animal types<br>• *E. coli* (pig, cattle)<br>• Campylobacter coli (pig)<br>Reporting content

No.	Country	EU Member State	Reporting to EFSA*	PPS extracted^	National level integrated surveillance (9) Comparison of report content and frequency of data reporting compared to EFSA reports
					-<br>Additional data available<br>Last published report contains 2019 data.
29	Slovenia	✓	✓		
30	Slovakia	✓	✓	✓	
31	The United Kingdom	✓	✓	✓	✓ VARSS<br>Pathogen & animal types:<br>• *E. coli* (chicken, turkey, pigs)<br>• *Salmonella* (chicken, turkey)<br>• *Campylobacter jejuni* (chicken, turkey)<br>Reporting content:<br>Animal types and pathogen in-line with EFSA requirements, with same sample size.<br>Additional data available:<br>Last published report contains 2020 data.

*Last published report in April 2022 contains data from 2019/2020
^Where at least one PPS extracted per country, either published or data collected between 2000 and 2021

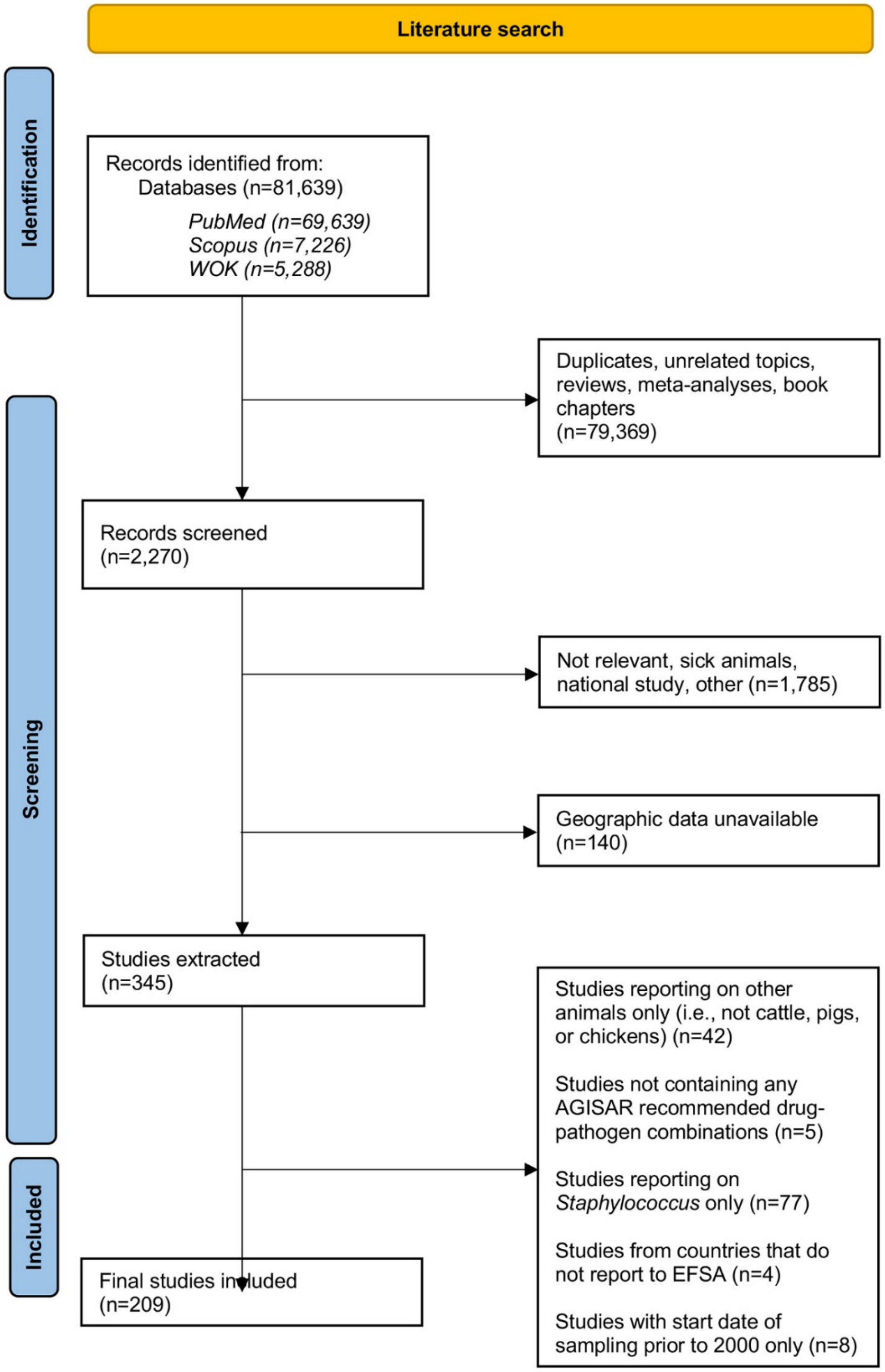

Appendix Figure 1. PRISMA Flow Diagram

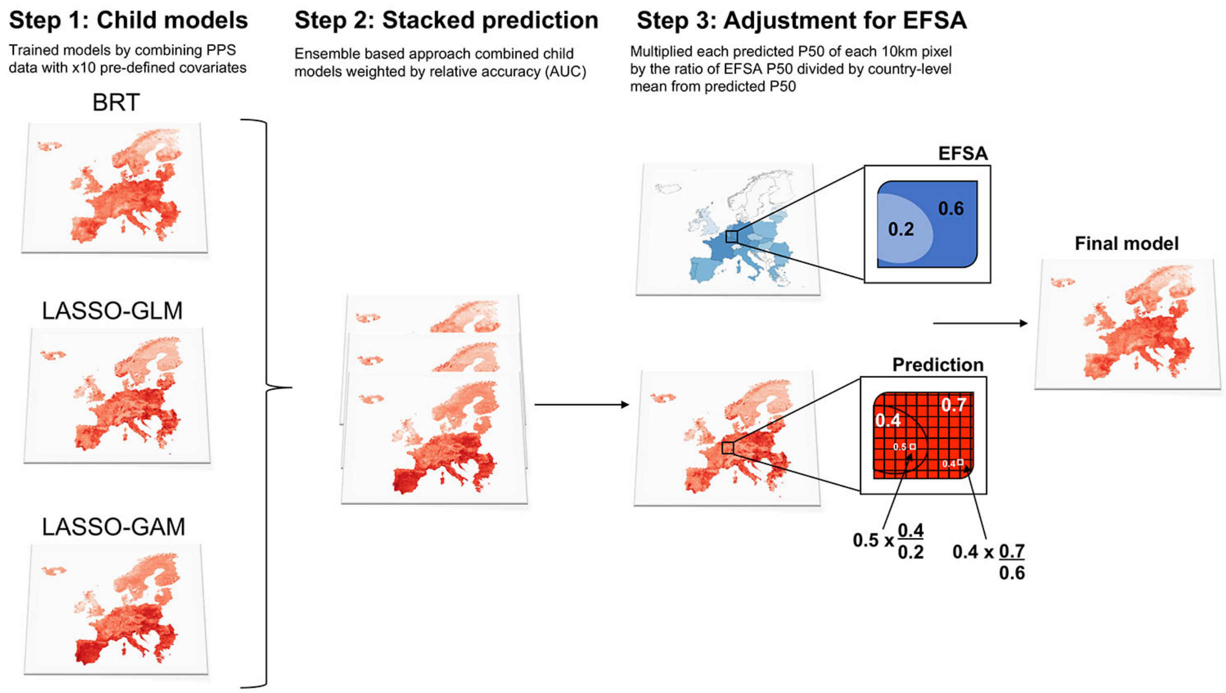

Appendix Figure 2. Geospatial modelling framework

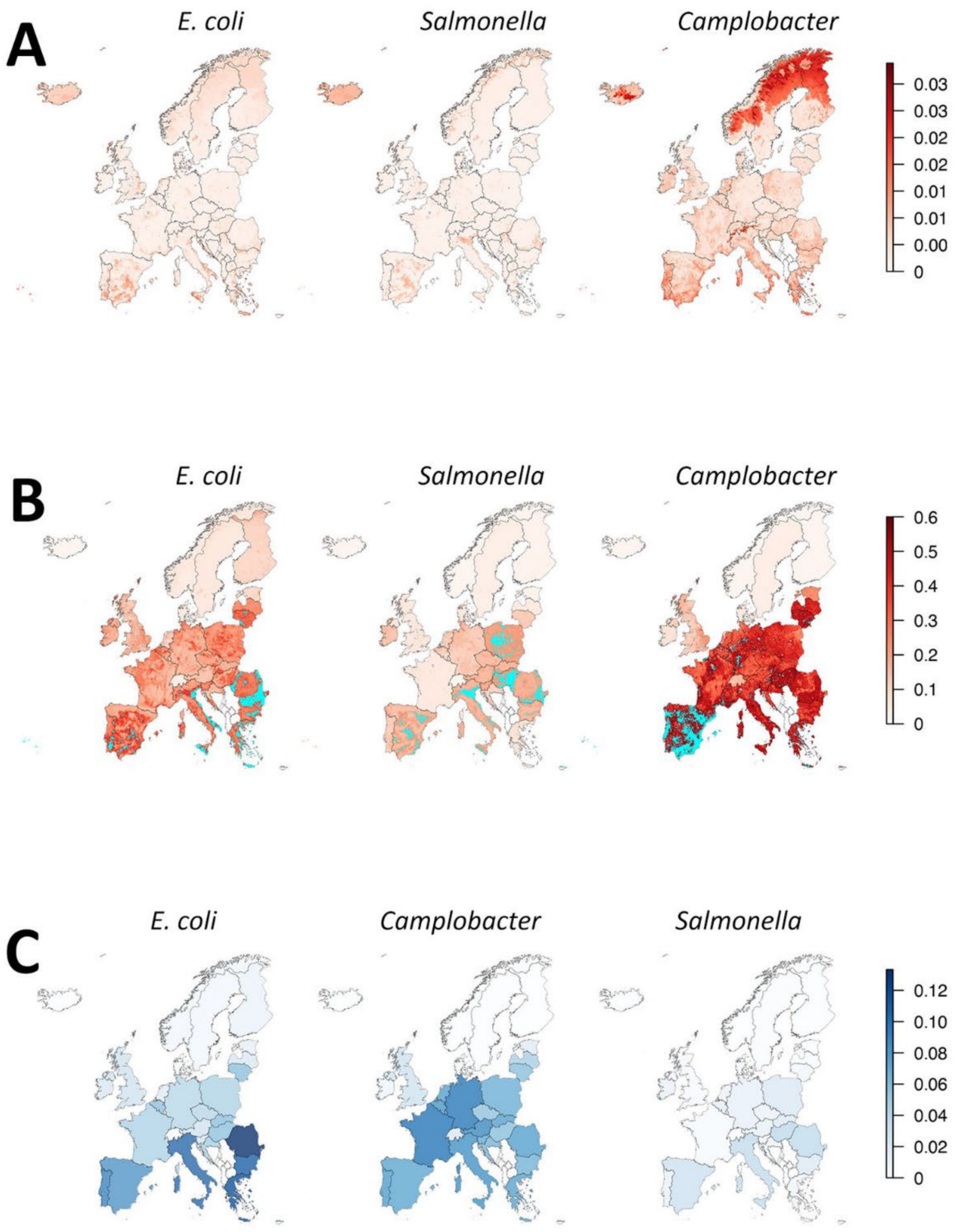

Appendix Figure 3. (a) Prediction uncertainty calculated from the variation of predicted P50 values across the ten bootstraps **(b)** Hotspot map for 31 countries (light blue indicates hotspot areas, the top 95% percentile) **(c)** Standard deviation in P50 estimates per country

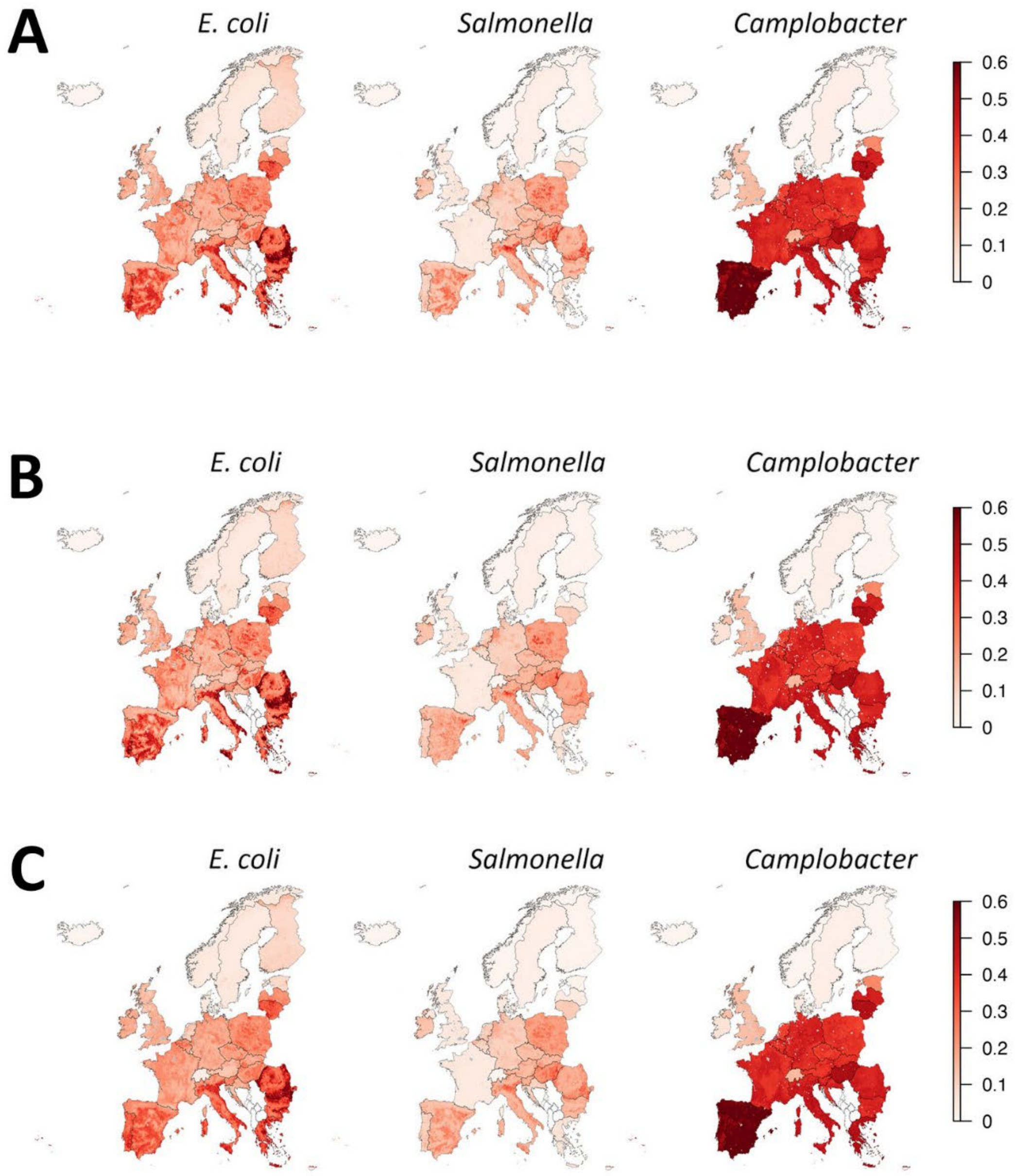

Appendix Figure 4. Sensitivity analyses of geospatial modelling for (a) date restriction to 2009-2020 only (b) 6-7 most common drug-bug combinations and (c) P50 by class of drug rather than individual compound.

References

1. Van Boeckel TP, Pires J, Silvester R, Zhao C, Song J, Criscuolo NG, et al. Global trends in antimicrobial resistance in animals in low- and middle-income countries. Science. 2019;365:eaaw1944. PubMed https://doi.org/10.1126/science.aaw1944

2. World Health Organization. Integrated surveillance of antimicrobial resistance in foodborne bacteria: application of a one health approach: guidance from the WHO Advisory Group on Integrated Surveillance of Antimicrobial Resistance (AGISAR). Geneva: The Organization; 2017.

3. Weiss DJ, Nelson A, Gibson HS, Temperley W, Peedell S, Lieber A, et al. A global map of travel time to cities to assess inequalities in accessibility in 2015. Nature. 2018;553:333–36.

4. Van Boeckel TP, Brower C, Gilbert M, Grenfell BT, Levin SA, Robinson TP, et al. Global trends in antimicrobial use in food animals. Proc Natl Acad Sci U S A. 2015;112:5649–54. PubMed https://doi.org/10.1073/pnas.1503141112

5. Fick SE, Hijmans RJ. WorldClim 2: new 1-km spatial resolution climate surfaces for global land areas. Int J Climatol. 2017;37:4302–15. https://doi.org/10.1002/joc.5086

6. Siebert S, Döll P, Hoogeveen J, Faures J-M, Frenken K, Feick S. Development and validation of the global map of irrigation areas. Hydrology and Earth System Sciences. 2005;9:53547, 200.

7. Gilbert M, Nicolas G, Cinardi G, Van Boeckel TP, Vanwambeke SO, Wint GRW, et al. Global distribution data for cattle, buffaloes, horses, sheep, goats, pigs, chickens and ducks in 2010. Sci Data. 2018;5:180227. PubMed https://doi.org/10.1038/sdata.2018.227

8. Hansen MC, Potapov PV, Moore R, Hancher M, Turubanova SA, Tyukavina A, et al. High-resolution global maps of 21st-century forest cover change. Science. 2013;342:850–3. PubMed https://doi.org/10.1126/science.1244693

9. Schrijver R, Stijntjes M, Rodríguez-Baño J, Tacconelli E, Babu Rajendran N, Voss A. Review of antimicrobial resistance surveillance programmes in livestock and meat in EU with focus on humans. Clin Microbiol Infect. 2018;24:577–90. PubMed https://doi.org/10.1016/j.cmi.2017.09.013
